# Harnessing Sun’s Energy with Quantum Dots Based Next Generation Solar Cell

**DOI:** 10.3390/nano3010022

**Published:** 2012-12-27

**Authors:** Mohammad A. Halim

**Affiliations:** Department of Chemistry & Chemical Biology, McMaster University, Hamilton, Ontario L8S 4M1, Canada; E-Mail: mhalim4@uwo.ca; Tel.: +1-519-661-2111 (ext. 86667).

**Keywords:** solar energy, light harvesting, quantum dots, cadmium sulfide, lead sulfide

## Abstract

Our energy consumption relies heavily on the three components of fossil fuels (oil, natural gas and coal) and nearly 83% of our current energy is consumed from those sources. The use of fossil fuels, however, has been viewed as a major environmental threat because of their substantial contribution to greenhouse gases which are responsible for increasing the global average temperature. Last four decades, scientists have been searching for alternative sources of energy which need to be environmentally clean, efficient, cost-effective, renewable, and sustainable. One of the promising sustainable sources of energy can be achieved by harnessing sun energy through silicon wafer, organic polymer, inorganic dye, and quantum dots based solar cells. Among them, quantum dots have an exceptional property in that they can excite multiple electrons using only one photon. These dots can easily be synthesized, processed in solution, and incorporated into solar cell application. Interestingly, the quantum dots solar cells can exceed the Shockley**-**Queisser limit; however, it is a great challenge for other solar cell materials to exceed the limit. Theoretically, the quantum dots solar cell can boost the power conversion efficiency up to 66% and even higher to 80%. Moreover, in changing the size of the quantum dots one can utilize the Sun’s broad spectrum of visible and infrared ranges. This review briefly overviews the present performance of different materials-based solar cells including silicon wafer, dye-sensitized, and organic solar cells. In addition, recent advances of the quantum dots based solar cells which utilize cadmium sulfide/selenide, lead sulfide/selenide, and new carbon dots as light harvesting materials has been reviewed. A future outlook is sketched as to how one could improve the efficiency up to 10% from the current highest efficiency of 6.6%.

## 1. Why Do We Need Renewable Solar Energy?

*“Sustainable Development is development that meets the needs of the present without compromising the ability of future generations to meet their own needs* *.”*

Our Common Future, Brundtland Report, 1987.

**Figure 1:**
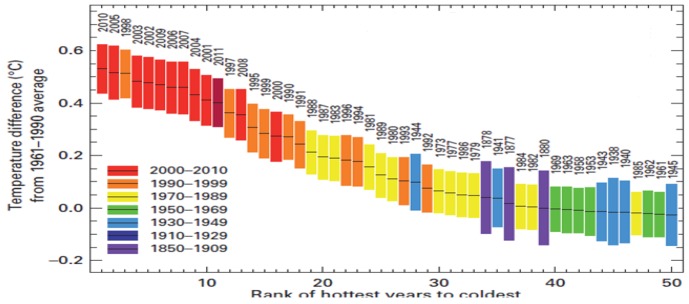
Global ranked surface temperature report 2011. Reproduced with permission from [3], Copyright 2012, World Meteorological Organization.

By disclosing the scientific facts of man-made climate change due to global warming and its impact on environmental and socio-economical systems, the Intergovernmental Panel on Climate Change (IPCC) made seminal contributions. Because of their contributions, they were awarded 2007 Nobel Peace Prize shared with Albert Arnold Gore, a former vice president of United States [1]. As many scientists were (and still are) very skeptical of global warming, in addressing this point IPCC made a bold statement: “*Warming in climate systems is unequivocal and most of warming due to green house gases*” [2]. The annual WMO (World Meteorological Organization) report revealed that 2010 was the warmest year in the record, and 2005 was the second warmest year as demonstrated in Figure 1 [3].Although the average temperature of 2011 did not demonstrate record-setting values like the other warmest years, it ranked the eleventh warmest year in the record. This finding unveiled that the 2000s (2000–2009) were warmer than the 1990s (1990–1999) which in turn were warmer compared to the 1980s (1980–1989) and earlier decades [3].

**Figure 2:**
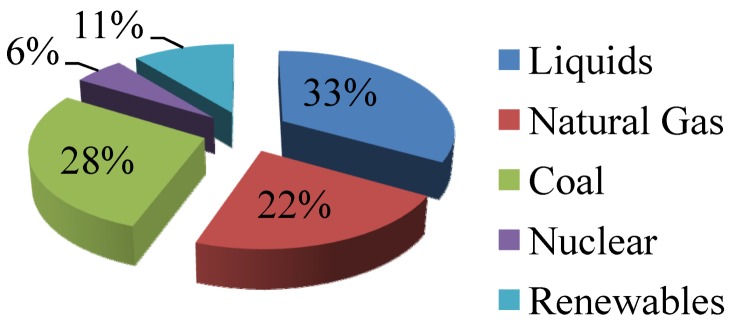
US Environmental Information Administration (EIA) global energy outlook 2011 [4].

The use of fossil fuels has been viewed as a major environmental threat because of their substantial contribution to greenhouse gases. The world energy consumption outlook 2011 which was published by the US Environmental Information Administration (EIA) disclosed that our energy consumption which relies heavily on the three components of fossil fuels (oil, natural gas and coal) is increasing despite some attempts incorporating other sources [4]. The report revealed that 83% of our present primary energy source was exploited from fossil fuels as depicted in Figure 2. Only 6% of our energy consumption of that year came from nuclear power. However, the recent massive earthquake and subsequent Tsunami in Japan resulted in an unprecedented nuclear disaster which raised many questions on public health and safety in the use of nuclear energy [5]. Although contributions from the renewable sources slightly increased since the previous years, these sources still contributed only 11% of our energy uses. The major challenge, therefore, in our energy sector is to increase the contribution from renewable energy sources. These alternative sources of energy need to be very abundant, environmentally clean, efficient, sustainable, renewable, safe, and cost effective. These conditions encourage many researchers to harness the sun’s energy which could not just solve the present energy problem but also fulfill our future demand. As one hour solar energy can be used for one year, therefore, we only need to harvest less than 0.02 of solar energy [6].

## 2. Many Approaches for Harnessing Sun’s Energy

*“I have no doubt that we will be successful in harnessing the sun* *’s energy. If sunbeams were weapons of war, we would have had solar energy centuries ago* *.”*

George Porter (1920–2002), Winner of 1967 Nobel Prize in Chemistry.

### 2.1. Silicon Based Solar Cells

There are many initiatives solving our energy problems and most efficient and popular one is the silicon based solar cells [7]. The lab based performance of silicon based solar cell has recently reached about 25%; however, market based efficiency is lower in the range of 15%–22.4%. In this year, the market based silicon solar panel produced by Suntech [8] has an efficiency of 15.7%; however, solar panel installed by the SunPower [9] has a record efficiency of 22.4%. Although silicon solar cells made by mono, multicrystalline, and amorphous thin films share about 85% of today’s market, the major cost factors related with silicon based solar cell include requirements of high purity silicon, high preparation temperature, and large amount of materials in order to prepare a tiny cell [10]. A report on cost profile of PV technologies disclosed that monocrystalline, multicrystalline, and amorphous silicon based solar panels cost $3.83, $3.43, and $3.00, respectively which are comparatively higher than other solar panels [11]. However, in a recent interview, Stuart Wenham, chief technology officer of the Suntech Power, claimed that the recent prize of a cell module is reduced to US$1.5 W^−1^ from the previous prize of US$4 W^−1^ [10]. Despite many challenges with silicon wafer based solar cells, it is expected that silicon based photovoltaic technology will be dominated in the future market.

### 2.2. Dye Sensitized Solar Cells

**Figure 3:**
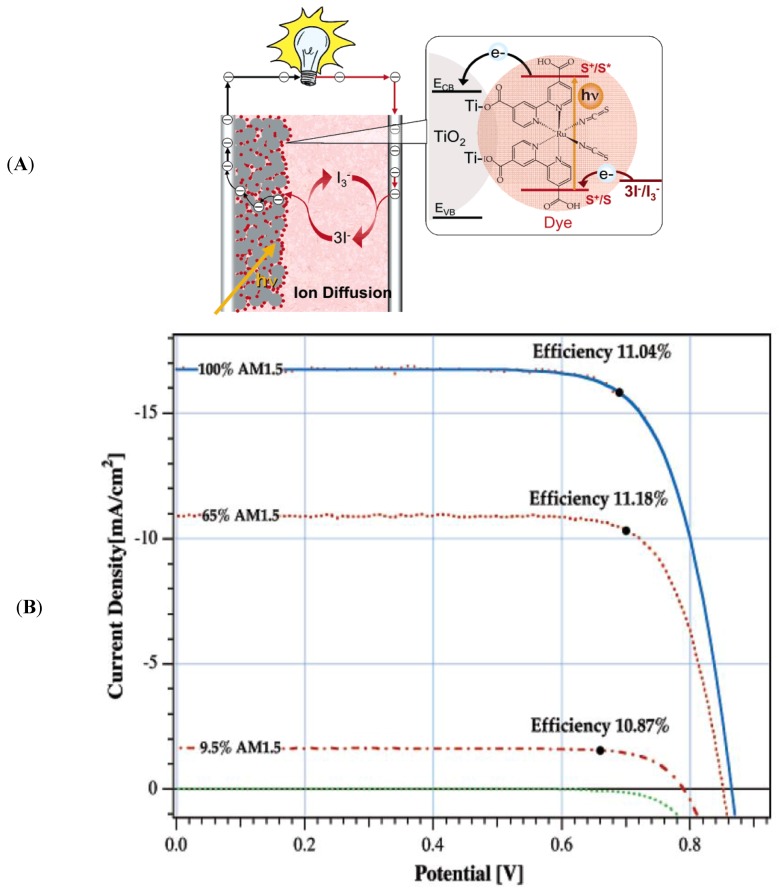
(**A**) The dye sensitized solar cell; (**B**) PV curves show the power conversion efficiency of dye sensitized solar cell in different light intensities. Reproduced with permission from [18], Copyright 2005, American Chemical Society.

**Figure 4:**
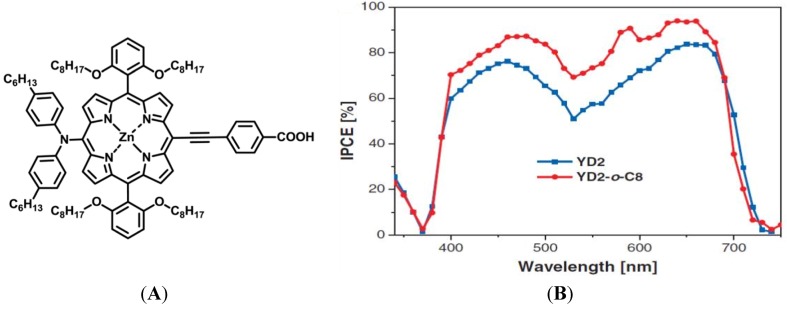
(**A**) The molecular structure of the donor-p-bridge-acceptor zinc porphyrin dye(YD2-o-C8); (**B**) IPCE performance of YD2-o-C8 and YD2 dyes. Reproduced with permission from [20], Copyright 2011, the American Association for the Advancement of Science.

Dye-sensitized solar cells (DSCs) invented by Michael Grätzel became a very popular alternative to silicon based solar cells because of their great potential to convert solar energy into electric energy at low cost [12,13,14,15,16]. This cell can be made from cheap materials such as inorganic and organic dyes which do not need to be highly pure as is required for silicon wafer [17]. The working principle of the solar cell is presented in Figure 3A. Here we can see inorganic dye is anchored to a wide bandgap mesoscopic semiconductor. The popular dyes used for DSC are ruthenium bipyridine and zinc porphyrin complexes. For a mesoscopic semiconductor, TiO_2_ (anatase) is widely used in the solar cells; however, other alternative metal oxides such as ZnO, SnO_2_ and Nb_2_O_5_ can be used. After excitation of dye by light, the dye releases its electron from the HOMO (highest occupied molecular orbital) to the LUMO (lowest unoccupied molecular orbital). This photoelectron then swiftly transfers from the LUMO of the dye to the conduction band of the semiconductor TiO_2_. The semiconductor carries the electron to the photoanode which passes the electron to the platinized counter electrode. Regeneration of the oxidized dye takes place by a redox couple such as iodide/triodide which reduces the dye by providing a continuous supply of electrons [18]. Over many years, the overall conversion efficiency of most solar cells was unchanged from 11.18% [19] as shown in Figure 3B. Only recently, Grätzel group was able to exceed the power conversion efficiency 12.3% [20]. In this research, they used donor-p-bridge-acceptor zinc porphyrin dyes of YD2-o-C8 and YD2 (see in Figure 4A) incorporating Co(II/III)tris(bipyridyl)–based redox couple instead of iodide/triiodide redox shuttle. The incident photon-to-electric current conversion efficiency of the YD2-o-C8 dye is relatively higher than the YD2 dye as depicted in Figure 4B. This study concluded that the incorporation of non-volatile electrolyte and new anchoring groups to the porphyrin dye increase the power conversion efficiency to 13%.

### 2.3. Organic Solar Cells

**Figure 5:**
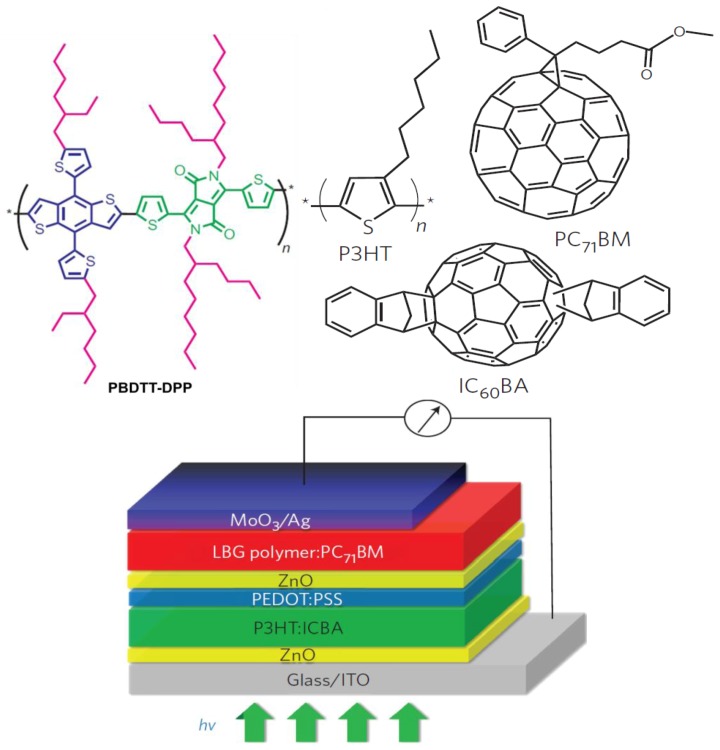
Molecular structures of PBDTT-DPP, P3HT, PC_71_BM and IC60BA (top). Tandem solar cell device (bottom).Reproduced with permission from [27], Copyright 2012, Macmillan Publishers Limited.

The seminal work of Heeger, Shirakawa and MacDiarmid (winners of 2000 Nobel Prize in Chemistry) opened a new window to use organic conducting polymer for wide range of semiconductor devices such as light emitting diodes, solar cells, and thin film transistors [21,22,23]. The motivation of developing organic materials for solar cell is to reduce the cost related to raw materials and manufacturing*. Solarmer* and *Konarka* Power Plastic*, *two US based companies, produce flexible polymer solar cells for many applications including portable electronics, smart fabrics, and integrated solar cells. The lab based power conversion efficiency of the polymer based single solar cells is reached to 8.6% reported by several groups [24,25,26,27]. Two well-known challenges associated with the donor-acceptor based polymer solar cell are that these polymers cannot cover the sun’s broad spectrum due to their comparatively high bandgap (1.6–2.0 eV) and they have lower carrier mobility. In order to exploit light from sun’s full spectrum, recently Dou *et al.* [27] developed a pyrrole (DPP) and dithiopene (BDT) based conjugated polymer poly{2,6'-4,8-di(5-ethylhexylthienyl)benzo[1,2-b;3,4-b]dithiophene-alt-5-dibutyloctyl-3,6-bis(5-bromothiophen-2-yl)pyrrolo[3,4-c]pyrrole-1,4-dione} (PBDTT-DPP) having a bandgap of 1.44 eV depicted in Figure 5 (top). This conjugated polymer has relatively higher carrier mobility. The tandem cell was constructed using Poly(3-hexylthiophene) (P3HT) and indene-C60 bis-adduct (IC60BA) as front-cell materials, and PBDTT-DPP together with the acceptor phenyl-c71-butyric acid methyl ester (PC71BM) as back-cell materials as shown in Figure 5 (bottom). Very recently, the solution phase tandem polymer based solar cell is achieved a record highest efficiency of 10.6% which is certified by NREL [28]. The life time of the polymer based solar cell (PSC) is comparatively low to 3–7 years which is one of the major challenges of PSC facing in market [29].

## 3. Breaking the Shockley-Queisser Limit

A major problem with single band gap solar cells including silicon wafer, inorganic dye, and polymer is that their efficiency cannot go beyond the Shockley-Queisser efficiency limit of 33% [30]. Theoretical calculation showed that 1.13 eV bandgap of single solar cell is lost 47% of the energy as heat when the hot electrons are moving down from the higher conduction band to the conduction band edge, 2% of energy is lost due the recombination when the tapped electrons from the conduction band is returned to the valence band, and 18% of energy is lost when photons failed to excite electrons which reside on the upper and lower bandgap compared to the 1.13 eV bandgap [7,31,32].

**Figure 6:**
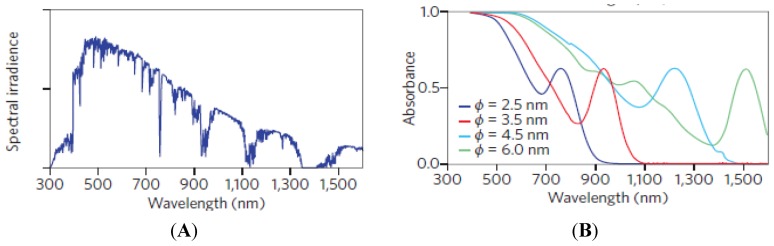
(**A**) Power spectrum of the Sun; (**B**) Changing size of the quantum dot one can shift the infrared wavelength to the blue wave length. Reproduced with permission from [36],Copyright 2012, Macmillan Publishers Limited.

Interestingly, a tiny material known as quantum dot can excite two or multiple electron-hole pairs (excitons) at a time while absorbing one photon [33]. This process is known as multiple exciton generation (MEG) for quantum dots and semiconductor nanocrystals because these electron-hole pairs are not free; however, they are confined (quantum confinement) in dimension due to their tiny size. A similar process (known as impact ionization) appeared in bulk semiconductors which requires relatively higher energy compared to semiconductor nanocrystals and quantum dots for producing multiple electron-hole pairs [34,35]. For example, impact ionization requires 7 eV (180 nm) incident photon energy for producing one extra electron-hole pair in silicon based bulk semiconductor [34,35]. That is why these bulk semiconductors based solar cells’ materials such as silicon, inorganic dyes, and organic polymers which easily excite one electron at a time are not ideal candidates for third generation photovoltaic technology. Theoretical studies revealed that the multiple exciton generation (MEG) based solar cells which utilized quantum dots and semiconductor nanocrystals can exceed the S-Q limit with an efficiency of 66% and even higher of 80% [7]. Moreover, changing the size of the of quantum dots one can utilize the Sun’s broad spectrum of visible and infrared ranges presented in Figure 6 [36]. Another advantage with quantum dots is that they can be processed in solution and can easily be incorporated into solar cell device [37,38].

**Figure 7:**
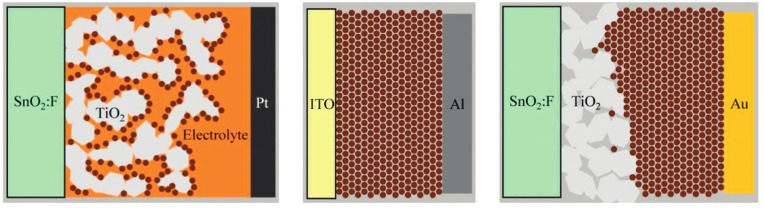
The graphical presentation of the QD sensitized solar cell, QD based Schottky solar cell, and QD based depleted heterojunciton solar cell (from left to right). Reproduced with permission from [39], Copyright 2010, American Chemical Society.

The quantum dot based solar cells vary with different architectures including QD sensitized solar cells, QD based Schottky junction solar cells, and QD based depleted heterojunction solar cells [39]. A graphical illustration of the three quantum dot based solar cell is presented in Figure 7. The working principle of the QD sensitized solar cells is same as the dye sensitized solar cells which are described in section 2.2. For constructing Schottky junction solar cell, *p*-type QD film is developed on the transparent ITO (indium tin oxide) conducting oxide, and a layer of evaporated metal is used for covering the QD film. In case of QD based depleted heterojunction solar cell, mesoporous TiO_2_ is used for covering the QD film with a layer of evaporated metal (mostly gold). For these solar cells, a depleting region is created. In depleted solar cell, the depleted region is appeared between the electron accepting TiO_2 _and the quantum dots, and the region is eventually reached to the side of TiO_2_. However, for the Schottky solar cell, the depleted region is built up on the side of *p*-type semiconductor quantum dots. Although TiO_2_ is widely used as a semiconductor in the depleted solar cells, other materials such as ZnO, amporhorus-Si, and fullerene can be used as alternatives. Recently, Luther *et al.* [40] reported the performance of ZnO incorporated with 1.3 eV PbS QDs with an overall power conversion efficiency of 2.94%. The advantages and disadvantages of these three solar cells are described in a recent perspective by Hetsch *et al.* [41].

Different types of quantum dots are available; however, cadmium and lead sulfide/selenide based quantum dots are frequently used for solar cell application. In this section, I focus my discussion mainly on cadmium and lead based quantum dots, and issues with the redox couple and electrodes. I present a brief overview on some other quantum dots such as CdTe, Cu_2_S, CuInS_2_, InAs and InAs/GaAS, and include a short discussion on recently developed carbon based quantum dot.

### 3.1. Cadmium Quantum Dots Based Solar Cells

Cadmium sulfide (CdS) and cadmium selenide (CdSe) are the two most studied quantum dots related to solar cell application. The reason for their widespread use is that they can be prepared easily and can be processed in solution. The first air stable bilayer cell of nanocrystalline CdTe/CdS exhibited a remarkable performance with an overall power conversion efficiency of 2.9% [42]. One earlier ultrafast absorption and emission study by Robel *et al*. [43] with CdSe quantum dot employing bifunctional surface modifier HS-R-COOH proved that CdSe can inject electron from its excited state to the mesoscopic TiO_2_. Although the cell exhibited the photon-to-charge carrier generation efficiency (IPCE) of 12%, the power conversion efficiency was less than 1%. A similar study with CdSe incorporating a cobalt (II/III) based redox system, Lee *et al*. [44] was able to improve the performance of IPCE to 36% as well as the overall power conversion efficiency to 1%. Replacement of TiO_2_ with semiconducting single-walled carbon nanotubes (SWCNTs), stacked-cup carbon nanotubes (SCCNTs), and fullerene (C60) was studied which demonstrated that nanotubes can capture electrons from CdSe quantum dots [45,46]. Although these inorganic organic hybrid solar cells can be assembled, they failed to give any reportable power conversion efficiency.

Recently, Shu *et al*. [47] developed a series of CdSe*_x_*S_(1__−*x*)_ based solar cells using TiO_2_ as photoelectron acceptor and Na_2_S as electrolyte by the successive ionic layer adsorption and reaction (SILAR) technique. By varying the ratio of selenide and sulphur, they were able to achieve the overall conversion efficiency of 2.27%. In this study, they prepared core shell quantum dots using CdSe*_x_*S_(1__−*x*)_/CdSe and were able to promote the efficiency to 3.17%. Similar studies conducted by Toyoda *et al*. [48] with CdS/CdSe solar cell using Na_2_S showed a slightly higher efficiency of 3.5%. Incorporating semiconductor SnO_2 _with CdS/CdSe solar cell improved the efficiency to 3.68% [49]. A study with different electrodes based CdS/CdSe solar cells disclosed that mixed CuS/CoS counter electrode can significantly increase the power conversion efficiency to 4.1% than the efficiency of the single electrode employing CuS (3.2%) and CoS (3.8%) [50]. However, the performance of the hollow core mesoporous shell carbon (HCMSC) counter electrode using polysulfide electrolyte was comparatively lower (1.08%) than the other electrolytes used in solar cells. Rod like CdSe quantum dots sensitized solar cells using ZnS electrode were reported recently and achieved an efficiency of 2.7% [51].

**Figure 8:**
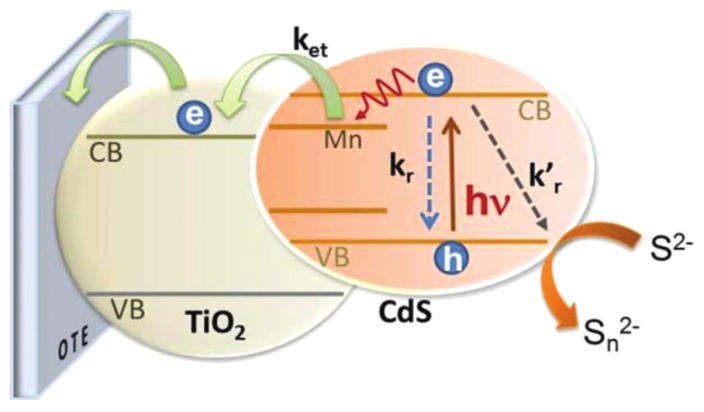
Mn-doped quantum dot based solar cell.Reproduced with permission from [52], Copyright 2012, American Chemical Society.

In a very recent study, Santra and Kamat [52] claimed to achieve an efficiency of 5.42% for a CdS/CdSe based solar cell fabricated by successive ionic layer adsorption and reaction (SILAR) approach presented in Figure 8. In this study, they used Mn as a dopant since it can modify the electronic and photophysical properties of the quantum dots and can create different electronic states in the intermediate regions. Forming intermediate band gaps can help to reduce the recombination loss of electron from TiO_2_ to the valence band of the quantum dots. In this study, it was observed that the absorption peak of the Mn-doped CdS quantum dots was increased to 570 nm (corresponding band gap 2.6 eV) relative to the peak at 520 nm (corresponding band gap 2.4 eV) for undoped CdS. Although hybrid quantum dots CdS/CdSe shifted the absorption peak further red to 690 nm; however, only a slight difference was observed between Mn-doped and Mn-undoped CdS/CdSe. In comparison with four quantum dots solar cells, the highest incident-photon-to-carrier conversion-efficiency (IPCE) is observed in the Mn-doped CdS/CdSe based solar cell with an enhancement value from 68% to 80%. The other important photovoltaic parameters including short circuit current (*Isc*), open circuit voltage (*Voc*), fill factor (*FF*), and power conversion efficiency (*η*) of the Mn-doped CdS/Se are 20.7 mA/cm^2^, 558 mV, 0.47 and 5.42%, respectively. 

Most of the QDSSCs are fabricated by direct growth approaches including chemical bath deposition (CBD) or SILAR where quantum dots are grown onto the thin film of a mesoporous TiO_2_ semiconductor [49,52,53,54,55,56,57,58]. In comparison with other methods, these direct growth methods are sought to be more effective since they can maintain high coverage of QDs and uniformly control the particle size and distribution on the surface of TiO_2._ However, some recent studies confirmed that utilizing other methods one can enhance the performance of the QDSSCs. Pan *et al*. [53] reported the fabrication of inverted type-I CdS/CdSe quantum dot solar cell by employing an organometallic high-temperature synthesis protocol where presynthesized quantum dots are assembled to the TiO_2_ using the mercaptopropionic acid (MPA) acting as a linker molecule. Under full sun illumination, this core/shell solar cell achieved a power conversion efficiency of 5.32%. Although the short circuit voltage (*Isc* = 18.02 mA/cm^2^), open circuit voltage (*Voc* = 527 mV) of this cell is relatively low compared to Mn-doped CdS/Se solar cell, the fill factor value is slightly high (*FF* = 0.56). Another method known as postsynthesis assembly where *ex situ* ligand exchange strategy is used for deposition of QDs onto TiO_2_ can also promote the performance of QDSSC. For instance, using this method Zhang *et al*. [59] were able to develop a record 5.42% efficient CdSe quantum dot based solar cell. This efficiency is exactly same as the Mn-doped CdS/Se solar cell fabricated by Santra and Kamat [52]. This postsynthesis approach exhibited high surface coverage (34%) and took 2 h for the deposition of QDs onto the TiO_2_. The *Isc* (16.96 mA/cm^2^), *Voc* (561 mV), and *FF* (0.566) values of the postsynthesized CdSe solar cell are comparable with the values of the SILAR based Mn-doped and presythesized CdS/CdSe solar cells.

### 3.2. Lead Quantum Dots Based Solar Cells

PbS quantum dot is an ideal material for solar cells which can be used as an electron donor for wide bandgap semiconductors including TiO_2 _and ZnO. PbS and PbSe quantum dots of group IV–VI have some exceptional properties including (i) efficient light absorbing capacity from visible and near IR regions, (ii) relatively long excitonic life time (200–800 ns), (iii) high quantum efficiency (80%), (iv) comparatively large Bohr radius (18nm of PbS and 46nm of PbSe), (v) water solubility [59,60,61,62]. In an earlier study, Plass *et al*. [63] showed that the power conversion efficiency of a PbS based heterojunction solar cell (prepared by chemical bath deposition method) incorporating organic charge transport material spiro-OMeTAD was less than 1% (more details about solid-state QDSSCs are discussed in section 4.). However, PbSe quantum dots using 1,2-ethanedithiol (EDT) organic ligand can improve the performance up to 2.1% [64]. Employing a new technique known as electrophoretic deposition for constructing colloidal PbS and PbSe solar cells linked with polysulfide electrolytes showed a low power conversion efficiency of 2.1% [65]. The mixed PbS*_x_*Se_1__−x _prepared with an one-pot hot injection reaction method demonstrated a higher power conversion efficiency of 3.3% which is comparatively higher than the efficiency observed for pure PbS and PbSe QDs based device [66]. By reducing the size to 2.3 nm of PbSe (band gap 1.6 eV) and using a modified one-pot hot injection method, Ma *et al*. [67] were able to promote the power efficiency to 4.7% with AM1.5 illumination. However, the open circuit voltage of this cell was initially enhanced by increasing the bandgap and the effect diminished earlier than expected. Further reducing the bandgap of colloidal PbS to 1.3 eV increased the average power conversion efficiency to 4.9%, and the champion device achieved an efficiency of 5.1% with *Voc* = 0.51 V, *Isc* = 16.2 mA cm^−2^, and *FF* = 58% [39]. Recently, for the first time Etgar *et al*. [68] used PbS quantum dots with anatase TiO_2_ nanosheets incorporating its dominant facet (001) to construct a heterojunciton solar cell using a simple hydrothermal protocol where tetrabutyltitanate and HF act as precursor and solvent, respectively. Under 0.9 light intensity, this cell achieved a power conversion efficiency of 4.73%.

**Figure 9:**
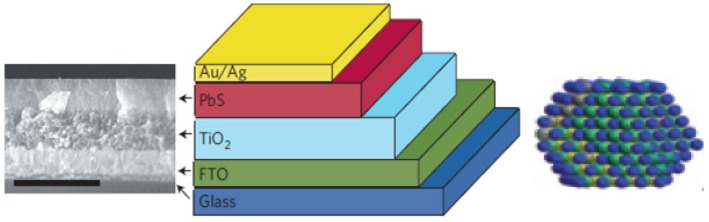
Colloidal quantum dot (CQD) based solar cell (middle) in which atomic passivation of QD is performed with inorganic halide ligand. SEM image in the left and Br^−^ligand passivated PbS CQDs in the right. Reproduced with permission from [70], Copyright 2011, Macmillan Publishers Limited.

The Sargent group in Toronto first constructed tandem colloidal quantum dots solar cells where they employed quantum dots having a bandgap of 1.6 eV for covering visible region (front cell) and another quantum dots of 1 eV for the infrared region (back cell) [69]. They used a TiO_2_ semiconductor to accept the photoelectron. In order to allow a barrier free transport of electrons from one junction to another junction, they employed a new approach named graded recombination level (GRL) using *n*-type MoO_3_, iridium tin oxide (ITO) and aluminum-doped zinc oxide (AZO). The highest power conversion efficiency of this two junction solar cell was 4.2%. However, the power conversion efficiency of the individual junction was about 3.0%. The short circuit current (*Isc*), open circuit voltage (*Voc*), and fill factor (*FF*) of the tandem was 8.3 mA cm^−2^, 1.06 V, and 48%, respectively. Although open circuit voltage of the tandem cell was comparatively higher, the fill factor value was relatively low. The low power efficiency of the tandem may be due to the large interparticle space between the quantum dots since organic ligands usually create space because of their long chain. Moreover, these organic ligands create insulating barrier between the colloidal quantum dots which eventually block the efficient electron-hole transport. However, some short organic and inorganic ligands can passivate the surface of quantum dots and can densify the films within the solid state. This process is known as “atomic ligand passivation”. In a recent study, the Sargent group used inorganic ligands which not only can minimize the interparticle space but also can promote the rapid electron-hole transport by passivating the surface of colloidal PbS QDs as shown in Figure 9. Inorganic halide ligands (Cl^−^, Br^−^, I^−^) lowered the recombination loss which is one of the reasons for lower power conversion efficiency. This inorganic ligand based colloidal QDs solid state solar cell promoted the power conversion efficiency to 6% which was the highest efficiency found in quantum dots based single solar cells in 2011 [70]. In 2012, this group was able to improve the power conversion efficiency (6.6%) of PbS colloidal based all inorganic (homojunction) quantum dots solar cell by introducing a new solution-phase halide passivation technique which promotes high carrier mobility [71].

### 3.3. Some Other Quantum Dot Based Solar Cells

Although CdTe has widely been used for thin film solar cells, the application of this compound for constructing quantum dot solar cell is less promising because the bandgap (1.54 eV), valence band (0.54 V) and conduction band (−1.0 V) energies of this dot are not suitable for absorbing the light of visible and near IR regions [38]. Despite having negative conduction band energy which promotes fast electron injection into TiO_2_, the external quantum efficiency of CdTe QDs is less than 3% whereas CdSe is 70% efficient [38]. Some applications of this material as quantum dots solar cells are reported where CdHgTe and CdTe QDs are deposited on TiO_2_ showing power conversion efficiencies of 1.0% and 2.2%, respectively [72]. The CdTe semiconductor nanocrystals are incorporated with functional conjugated polymer of monoaniline-capped poly[(4,4'-bis(2-ethylhexyl)-dithieno[3,2-*b*:2',3'-*d*]silole)-2,6-diyl-*alt*-(2,1,3-benzothiadiazole)-4,7-diyl] (PSBTBTNH_2_) and obtained a conversion efficiency of 3.2% [73]. Some thin film solar cells fabricated with CdTe nanocrystals showed comparatively better performance. For instance, a simple Schottky diode of ITO/CdTe/Al achieved a power conversion efficiency of 5.15% [74] and CdTe/ZnO thin film system showed an efficiency of 6.9% [75].

Copper based quantum dots including CuS_2_ and CuInS_2_ (Copper Indium disulfide) are promising candidate for solar energy conversion devices due to their low toxicity, long term stability, low cost, better absorption efficiency (α = 5 × 10^5^ cm^−1^), and facile fabrication through various methods including (but not limited to) thermal, photochemical, microwave assistant decomposition of single source precursors, solvothermal, and SILAR [76]. Several studies have been focused to enhance the performance of the copper based quantum dots solar cell; however, the power conversion efficiency of these devices is failed to exceed 3%. A recently developed Cu_2_S-CuInS_2_-ZnSe based solar cell fabricated by the successive ionic-layer absorption and reaction approach (SILAR) was able to reach the efficiency of 2.52% [77]. An earlier study of colloidal CuInS_2_ quantum dots sensitized solar cell prepared without employing organic solvent achieved a lower efficiency of 1.47% [78]. Stability of the QDSSCs is a major concern associated with the common dots including PbS, CdS and CdSe. However, the stability of the InAs quantum dots based solar devices is quite remarkable and reproducible. Yu *et al*. [79] reported that in lower solar illumination intensity the power conversion efficiency of InAs QDSSC interfaced with Co redox system is approximately 1.7%, and unfortunately the efficiency is declined to 0.3% at high light intensity. Recently, Tanabe *et al*. [80] demonstrated that a five layered InAs/GaAs quantum dot solar cell fabricated by metalorganic chemical vapor deposition (MOCVD) method which suppresses the open circuit voltage (*Voc*) degradation can drastically increase the efficiency to 18.7% for 1 sun and 19.4% for 2 suns. Flexible plastic based InAs/GaAs QDSSC is also prepared by employing a bond-and-transfer method and the performance of this cell is quite significant showing an efficiency of 10.5% [81].

### 3.4. Carbon Dot Based Solar Cells

**Figure 10:**
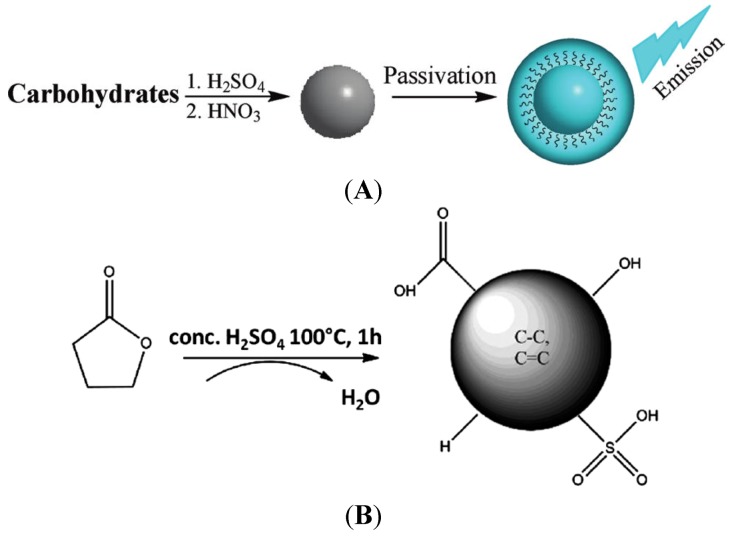
(**A**) Synthesis protocol of carbogenic dot. Reproduced with permission from [82], Copyright 2009, American Chemical Society. (**B**) Carbon dots prepared by Ozin group at Toronto (bottom). Reproduced with permission from [85], Copyright 2012, Royal Society of Chemistry.

It is obvious that metal based quantum dots are expensive and very vulnerable for health and environment. As a long term research goal, it would more appropriate if we can use other non-hazardous materials. In a previous study, Peng and Travas-Sejdic [82] synthesized luminescent carbogenic dots using carbohydrate as precursor materials presented in Figure 10A. This carbon dot first passivated by amine-terminated compounds, and due to less photoluminescence further passivation was performed with 4,7,10-trioxa-1,13-tridecanediamine (TTDDA). However, this carbogenic dot was not used for solar cell application. Highly soluble black graphene quantum dot have been prepared using a solubilization technique where polyphenylene dendrimer acts as a precursor [83]. This dot is used for sensitizing the TiO_2_ and comparable results are observed for short circuit current (*Isc* = 20 mA/cm^2^), open circuit voltage (*Voc* = 480 mV) and fill factor (*FF* = 0.58). Multiple exciton generation (MEG) is observed and collected in single-walled carbon nanotube based semiconductor; however, substantial future studies are required to integrate this material for solar cell application [84]. Recently, the Ozin group in Toronto was able to synthesize the carbon dots by dehydration of the γ-butyrolactone precursor depicted in Figure 10B [85]. Carbon dot can be emerged as a potential candidate in quantum dots based solar cells as sources of carbon are very versatile compare to metal based QDs. They were able to sensitize TiO_2_ with their carbon dots. However, the efficiency of the carbon dots based solar cell is only 0.13%. In this case, they used an I^−^/I_3_^−^ redox couple which is certainly not a good choice. Improving the efficiency of carbon dots based single solar cell requires finding out an optimal condition in case of size of quantum dots, ligands, electrolytes, and electrodes through comprehensive studies. In addition, carbon dots based tandem solar cell will be an ultimate choice in order to promote efficiency so that it can compete with other competitors.

## 4. Redox Couple and Counter Electrode

**Figure 11:**
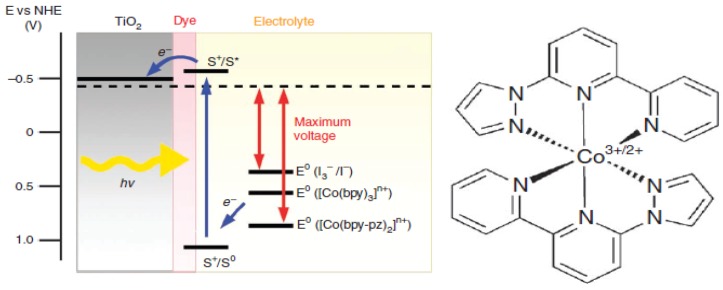
Dye sensitized solar cell using Co(III)/Co(II) based redox couple. Reproduced with permission from [92], Copyright 2012, Macmillan Publishers Limited.

As redox couple has an important role in dye and quantum dots sensitized solar cells, mostly iodide/triiodide redox couple is frequently used. However, this redox shuttle is not stable and is corrosive which is a threat to the working electrode, and also it gives a low open circuit voltage and fill factor [52,86]. There are many other redox couples available such as polysulfide (S^2^^−^/S*_x_*^2^^−^), ferrocene (Fc^+^/Fc), nickel [Ni(II)/Ni(III)], tetramethylthiourea, and cobalt [Co(II)/Co(III)] [87,88,89,90]. Ionic liquid base redox couple is also a promising candidate as it is compatible with quantum dot based sensitizers such as CdS, CdSe, PbS and PbSe. A recent study revealed that pyrrolidinium ionic liquid based electrolytes containing S^2^^−^/S*_n_*^2^^−^ redox couple can achieve an efficiency of 1.86% with CdSe quantum dot sensitized solar cell [91]. However, among these redox couples, the cobalt redox couple is stable and very facile for tuning the redox potential. This cobalt redox couple does not require an intermediary step during electron donating process related to quantum dot regeneration, and it can increase the open circuit voltage [92]. Redox potential of the electrolytes needs to be close to the quantum dots redox levels in order to increase the open circuit voltage (*Voc*) and rapid regeneration of the oxidized quantum dots [17]. Recently, Yella *et al*. [20] showed that Co(II/III)tris(bipyridyl) redox couple can increase the power efficiency of the dye sensitized solar cell from 11.18% to 12.4%. Interestingly, redox potential of the tridentate cobalt [Co(bpy-pz)_2_]^3+/2+^ (PF_6_)_3/2_ complex is 0.86 V versus NHE which is comparatively higher than the redox potential of I^−^/I_3_^−^ (0.37 V *versus* NHE) and Fc^+^/Fc (0.67 V *versus* NHE) depicted in Figure 11 [92].

Despite achieving the record highest efficiency of dye sensitized solar cell utilizing liquid electrolytes, there are some pivotal problems associated with these electrolytes which include solvent evaporation, leakage, corrosion, and short term stability [93,94]. These issues have been addressed first by Bach *et al*. [95] and they incorporated solid-state hole transport materials (HTMs) for solar cell application. Although various inorganic *p*-type semiconductors, organic materials, and conducting polymers can be used as hole transport materials; however, spiro-OMeTAD (2,2',7,7'-tetrakis-(*N*,*N*-di-p-methoxyphenylamine)-9,9'-spirobifluorene) is the most popular, efficient, and widely studied one [94]. This spiro-OMeTAD has many interesting properties including highly amorphous structure, easily soluble nature, relatively greater charge carrier mobility, high absorption capacity, and comparatively higher glass transition temperature [94,95,96]. Incorporating this organic hole transporter with Z907 dye and TiO_2_-coated ZnO in solid-state DSC provided a power conversion efficiency of 5.65% [97]. Mostly the spin-coating approach is widely used for the infiltration of spiro-OMeTAD in the DSC. This spin-coating technique requires greater amount of materials and is not suitable for filling the large device area whereas a relatively new roll-to-roll and large area coating technique known as doctor blading can be employed for infiltrating the spiro-OMeTAD in solid-state DSC [98]. Although this approach requires less material for pore filling, the performance of power conversion efficiency is relatively lower (3%) compared to the spin-coating technique [98]. One common shortcoming of the pure organic hole transporters is that they have low conductivity. A recent study in addressing this issue unveiled that Co(III) based *p*-type dopant can significantly increase the performance of the spiro-OMeTAD in solid-state dye sensitized solar cell with a power conversion efficiency of 7.2% [99].

The concept of solid-state hole transporters is also applied for quantum dot sensitized solar cells incorporating PbS and CdS. A study conducted by Lee *et al*. [100] disclosed that spiro-OMeTAD is compatible with PbS and CdS quantum dots; however, the challenge remains in improving the power conversion efficiency. The hybrid CdS/Squarine based solar cell achieved an efficiency of 1.2%, whereas the non-hybrid PbS solar cell is obtained an efficiency of 1.46% [100]. For this study, they employed successive ionic layer adsorption and reaction (SILAR) method for growing the quantum dots on the mesoporous TiO_2_. Yu *et al*. [101] introduced the polyacraylamide polymer matrix and polysulfide hydrogel electrolytes for constructing the quasi-solid-state CdS/CdSe sensitized solar cell, and this cell achieved an efficiency of 4%. Another CdS and CdSe quantum dots based quasi-solid-state solar cell with ZnO nanocrystalline film and polysulfide redox couple slightly promoted the efficiency to 4.5% [102]. One promising hole transporter material such as 3,3'''-didodecyl-quaterthiophene is also employed for preparing the all-solid-state QDSCs incorporating TiO_2_/CdSe; however, the power conversion efficiency is only 0.34% [103].

**Figure 12:**
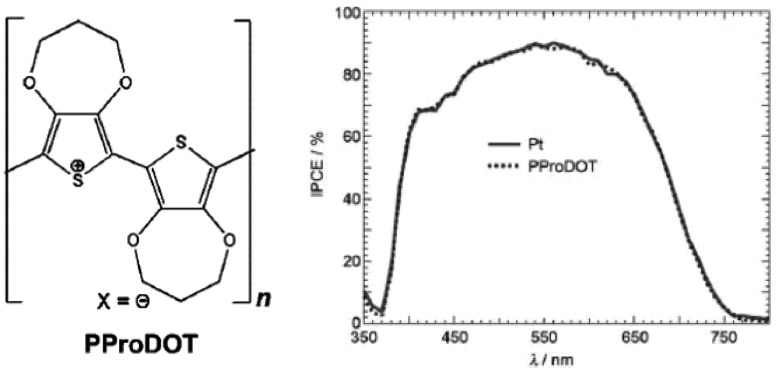
Molecular structure of PProDOT electrode (left) and IPCE performance of Pt and PProDOT electrode (right). Reproduced with permission from [105], Copyright 2010, Wiley-VCH Verlag GmbH & Co. KGaA, Weinheim.

Platinum and gold counter electrodes are very expensive; recently, other electrodes such as CoS, Cu_2_S, CuS/CoS and PProDOT [poly(3,4-propylenedioxythiophene)] electrodes are available to replace Pt and Au [50,104,105]. The molecular structure of the PProDOT electrode is presented in Figure 12 (left) and the performance of this electrode is comparable with the Pt electrode shown in Figure 12 (right) [105]. It is observed that cobalt [Co(bpy-pz)_2_]^3+/2+^ (PF6)_3/2_ redox couple is not appropriate for Pt electrode; however, combination of PProDOT electrode and the cobalt redox couple can promote the power conversion efficiency. It is, therefore, realized that the carbon based electrode can make stable, flexible, and cost-effective quantum dots based solar cells.

## 5. Photoinduced Electron Transfer

The performance of the solar cell relies on the smooth electron transfer and recombination processes involved in many parts of the solar cell including from the quantum dot to the semiconductor, from counter electrode to redox couple, from redox couple to the oxidized dot and vice versa [106]. Understanding the electron transfer and recombination processes can clearly help us to improve the solar cell device performance. In 1956, Rudolph Marcus, winner of the 1992 Nobel Prize in Chemistry, modeled the electron transfer occurring in two states [107]. This model was then extended for the electron transfer involved in semiconductor based dye-sensitized system which can be expressed as follows [108,109]:

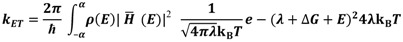


**Figure 13:**
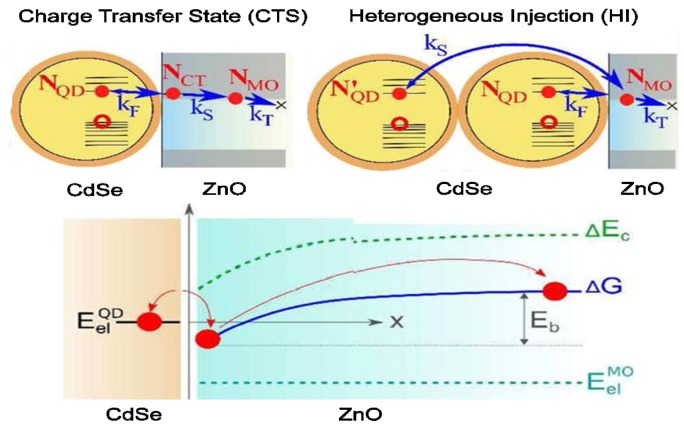
Charge transfer state (CTS) and heterogeneous injection models in the QD-MO system. Reproduced with permission from [112], Copyright 2012, American Chemical Society.

Here, *k*_ET_ is the electron transfer rate, *ħ* is the reduced Plank’s constant, *k*_B_ is the Boltzmann’s constant, λ is the total reorganization energy comes from inner and outer reorganization energies, ∆G is the free energy, ρ(E) is the density of the accepting states, and 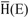 (E) is the overlap matrix element. By using this equation, one can calculate the electron transfer processes in the different parts of the solar cell employing constrained real-time time dependent density functional theory (C-RT-TDDFT) developed by Schatz group [110,111]. In most cases, time-resolved transient absorption (TA) spectroscopy and photoluminescence measurement have been employed for experimentally calculating the electron transfer rate between QDs to metal oxide (MO) [109,112]. However, one recent and interesting finding revealed that these two techniques can not provide the complete scenarios of the rapid and complex electron transfer process [112]. They proposed combined TA and time-resolved terahertz (THz) spectroscopy to study the electron transfer process in which TA can provide information of excited electrons in QDs and THz can aid to see the mobility of electron-hole pairs and excited electrons in MO. In this study, they used CdSe as QDs and ZnO nanowire as an electron acceptor. Their results summarized that the electrons are transferred from QDs to MO on a picoseconds time frame (τ = 3–12 ps) which confirmed that this transfer process is very fast compared to the loss of the excited electrons (Auger recombination). This finding supported that the injection of excited electrons from QDs to ZnO occurred through an intermediate charge-transfer state (CTS) and less likely with heterogeneous injection (HI) process as demonstrated in the Figure 13.

## 6. Future Outlook

*“I’d put my money on the sun and solar energy. What a source of power!!! I hope we don’t have to wait till oil and coal run out before we tackle that…”*

Thomas Edison (1847–1931)

The power conversion efficiency of different solar cells is presented in Table 1. There are two main goals for the next generation solar cell: (1) exceeding the Shockley-Queisser (S-Q) limit of 33% for the power conversion efficiency (2) reducing the price to a level of cents per kWh of electrical energy [33]. Theoretically, it is already proven that multiple exciton generation based certain quantum dots, metal based thin films (CdS, CdTe, CuInS_2_), and tandem GaInP/GaInAs/Ge solar cells can overcome the S-Q limit [7,113,114]. However, in reality the best performance of the QDSSCs remained on the level of efficiency around 5%–6%. Different fabrication techniques (chemical bath deposition, successive layer deposition, presynthesize, postsynthesize, one-pot hot injection, hydrothermal, ligand passivation, graded recombination layer) have been employed in order to enhance the performance of the QDSSCs. Some lead and cadmium based quantum dots solar cells are relatively more efficient compared to other quantum dots. Although numerous problems are involved with liquid electrolyte based solar cells, the highest power conversion efficiency of the liquid electrolytes based QDSSCs is reached to 6.6% whereas the efficiency of the solid-state QDSSCs is lacked behind to 4.5%. Another problem associated with single QD based solar cell that tiny quantum dots cannot absorb much light [31]. Solving this problem, we require incorporating several layers of quantum dots; however, connecting multiple layers together creates structural damage which might lower the efficiency. The available two-junction quantum dots solar cells developed by the Sargent group achieved a power conversion efficiency of 4.2% which is even lower than the efficiency (5.42% and 6.6%) obtained by the single quantum dot solar cells in different studies [52,69,71]. Despite numerous challenges, it is expected that multi-junction colloidal PbS and CdS quantum dots based solar cells can increase the power conversion efficiency up to 10% from the current efficiency 6.6%. It is observed that redox couples and electrodes can also enhance the performance of the solar cells. For example, in the case of dye sensitized solar cell the Co(II/III)tris(bipyridyl) redox couple was able to increase the power conversion efficiency from 11.18% to 12.3% [20]. Co(II/III)tris(bipyridyl) redox couple and platinum-free carbon based PProDOT counter electrode can be incorporated to quantum dot solar cell for promoting efficiency and reducing the cost. In future, more experimental attentions are required towards (i) improving the surface coverage of QDs onto the TiO_2_ for increasing the light harvesting efficiency of QDs, (ii) enhancing the electron and hole transfer rate and minimizing different recombination losses, (iii) improving the external and internal quantum yield in the visible and near-IR regions, (iv) increasing the high electron mobility through different types of electrodes by making nanopores, nanowires and nanopillars, (v) incorporating plasmonic interactions in the quantum dots, (vi) reducing the cost by improving the device performance of carbon based quantum dot solar cells [36,41,115].

**Table 1:** The power conversion efficiency of different solar cells.

Solar cells	Materials	Efficiency	References
Silicon	Si wafer	15.70%	[8]
		22.40%	[9]
Dye Sensitized	Ru bipyridine	11.18%	[18]
	Zn porphyrin	12.30%	[20]
Solid-State DSC	Z907 dye with spiro-OMeTAD (doctor-blading)	3.00%	[98]
	Z907 dye with spiro-OMeTAD	5.65%	[97]
	Y123 dye with spiro-OMeTAD and *p*-type Co(III) dopant	7.20%	[99]
Polymer	P3HT, IC60BA, PBDTT-DPP, PC71BM	8.60%	[27]
	Same as above ^a^	10.60%	[28]
Quantum Dots (Liquid Eletrolytes)	Rod like CdSe	2.70%	[51]
	CdSe_x_S_(1−*x*)_/CdSe	3.17%	[47]
	CdS/CdSe	3.50%	[48]
	CdS/CdSe	3.68%	[49]
	CdS/CdSe	4.10%	[50]
	CdS/CdSe	5.42%	[52]
	CdS/CdSe Invert Type I	5.32%	[53]
	CdSe	5.42%	[59]
	PbS/PbSe	2.10%	[65]
	Mixed PbS*_x_*Se_1__−*x*_	3.30%	[66]
	PbS (Tandem)	4.20%	[69]
	PbSe	4.70%	[67]
	PbS (TiO_2_ nanosheets)	4.73%	[68]
	PbS	5.10%	[39]
	PbS	6.00%	[70]
	PbS	6.60%	[71]
Solid State QDSSCs	CdSe with quaterthiophene	0.34%	[103]
	CdS/Squarine (Hybride)	1.20%	[100]
	PbS (Non-hybride)	1.47%	[100]
	CdS/CdSe with Polymer Matrix	4.00%	[101]
	CdS/CdSe/ZnO	4.50%	[102]

^a^ Detail information is not yet available.
